# Amorphization of Drugs for Transdermal Delivery-a Recent Update

**DOI:** 10.3390/pharmaceutics14050983

**Published:** 2022-05-03

**Authors:** Bappaditya Chatterjee, Abhishek Reddy, Moushami Santra, Sandile Khamanga

**Affiliations:** 1 Shobhaben Pratapbhai Patel School of Pharmacy and Technology Management, SVKM’s NMIMS, Mumbai 400056, India; bdpharmaju@gmail.com (B.C.); abhishekreddy.nmims2021@gmail.com (A.R.); moushamisantra.nmims2021@gmail.com (M.S.); 2 Faculty of Pharmacy, Rhodes University, Makhanda 6140, South Africa

**Keywords:** supersaturation, amorphous, transdermal, microneedle, controlled release, skin permeation

## Abstract

Amorphous solid dispersion is a popular formulation approach for orally administered poorly water-soluble drugs, especially for BCS class II. But oral delivery could not be an automatic choice for some drugs with high first-pass metabolism susceptibility. In such cases, transdermal delivery is considered an alternative if the drug is potent and the dose is less than 10 mg. Amorphization of drugs causes supersaturation and enhances the thermodynamic activity of the drugs. Hence, drug transport through the skin could be improved. The stabilization of amorphous system is a persistent challenge that restricts its application. A polymeric system, where amorphous drug is dispersed in a polymeric carrier, helps its stability. However, high excipient load often becomes problematic for the polymeric amorphous system. Coamorphous formulation is another approach, where one drug is mixed with another drug or low molecular weight compound, which stabilizes each other, restricts crystallization, and maintains a single-phase homogenous amorphous system. Prevention of recrystallization along with enhanced skin permeation has been observed by the transdermal coamorphous system. But scalable manufacturing methods, extensive stability study and in-depth in vivo evaluation are lacking. This review has critically studied the mechanistic aspects of amorphization and transdermal permeation by analyzing recent researches in this field to propose a future direction.

## 1. Introduction

It is beyond any doubt that the oral route is still the most accessible and popular mode of drug administration. However, many drugs cannot be effectively delivered via the oral route due to their inadequate oral bioavailability and instability in the gastrointestinal tract. The low oral bioavailability of drugs occurs mainly due to three reasons; poor aqueous solubility and dissolution of the drug (BCL class II and IV drugs, for example, telmisartan, ibuprofen, etc.), low intestinal permeability (BCS class II and IV drugs) and high first-pass metabolism (Example, raloxifene, artetether). A considerable percentage (40–50%) of therapeutically active drugs in the market belong to Biopharmaceutical class II and IV. Also, over 70% of new chemical entities under investigation are estimated to have poor water solubility. Several techniques have been adopted by the researchers and pharmaceutical industries to improve aqueous solubility of drugs, such as micronization or particle size reduction [1], cyclodextrin complexation [2], soluble salt formation [3], cocrystal formation [4], nanofibre [5], amorphous solid dispersion [6], etc. Drugs with low solubility and high permeability (BCS II) could be effective targets of these methods.

However, such techniques would not be quite effective for the drugs with high first-pass metabolism or gastrointestinal degradation. Commonly the first-pass effect is associated with the metabolism by the liver, but it may also happen in vasculature, lungs, gastrointestinal tract, and other metabolically active organs. For the drugs with high first-pass metabolism, regardless of poor or freely soluble, oral delivery is challenging because a significant fraction of the delivered and absorbed dose gets metabolized by the liver. Hence, the availability of the drug in systemic circulation becomes low. High first-pass and variable rate of liver metabolism may cause irregular systemic availability of a drug. Lipid-containing formulations such as self-emulsifying drug delivery have been demonstrated as a system that undergoes lymphatic transport after oral administration bypassing first-pass metabolism [7]. However, administrations other than oral are the best possible alternatives for high first-pass metabolism drugs. In such a context, transdermal route of drug delivery bears extreme importance and has gained notable popularity among formulation researchers. Apart from avoiding first-pass metabolism, transdermal delivery enjoys the advantages of easy and painless application, controlled drug release, the possibility of zero order drug release, avoidance of hostile gastric environment [8]. But, adequate permeability of the drugs through the skin by crossing the stringent barrier of the stratum corneum is the biggest challenge. High molecular weight and high hydrophilicity or lipophilicity of drugs are considered negative towards skin permeation after transdermal application.

Modified formulations such as nanoemulsions or microemulsions [9], solid lipid nanoparticles, nano lipid carriers [10], transfersomes [11], ethosomes [12], etc. are employed frequently to promote drug transport across the skin. In this review, another formulation approach has been focused on, which is an amorphous drug-containing system. In this system, the drug is dispersed either as molecular dispersion or in an amorphous state in a vehicle or carrier matrix, preferably hydrophilic. Solid dispersion, more specifically amorphous solid dispersion (ASD) has been successfully employed in oral formulation to enhance the bioavailability of poorly soluble drugs [13,14]. However, the concept of amorphous drug dispersion can be applied to other routes of drug delivery apart from oral, like in transdermal drug delivery. The idea of combining amorphous drugs and transdermal delivery has been experimented by some researchers for better drug permeation and controlling the drug release [15]. Loading of amorphous drugs into a transdermal system acts on the principle of supersaturation. While solvent evaporation is the most common technique of preparing a supersaturated transdermal delivery system [16], reducing organic solvent usage also increases. An amorphous drug-loaded system designed by hot melt extrusion could be a possible alternative. For the first time in this review, a critical understanding of how transdermal delivery can benefit from amorphization of drugs is presented. Both polymeric amorphous and coamorphous drug-loaded transdermal systems have been analyzed, and the relevant benefits and challenges are presented. The clinical and patent status of such systems has been studied. Finally, we have proposed a future direction for the relevant research.

## 2. Overall Features of Transdermal Drug Delivery

Transdermal Drug Delivery System (TDDS) is an alternative approach that allows the drug to permeate through skin and enter the systemic circulation, minimizing or avoiding the limitations of oral and parenteral formulations. In the subsequent section, highlights of various aspects of transdermal drug delivery and formulations have been presented in a summarized manner. Readers can go through extensive reviews on the scope and challenges of transdermal delivery published by other authors [17,18,19].

### 2.1. Skin as a Barrier to Transdermal Drug Delivery

The primary role of stratum corneum (SC) is protecting our body from external potentially harmful substances. However, such a natural protective barrier restricts the systemic entry of most therapeutic agents except those with fair lipophilicity (log P > 1.5) and molecular weight < 500 Da. The stringency of the SC is originated from the tightly bound, rigid keratinocytes cell. These ‘dead’ cells consist of keratin filaments and various cross-linked proteins [20,21,22]. Due to their strong barrier nature, keratinocytes are popularly known as the “brick-mortar” layer, where ‘mortar’ is the surrounding intracellular multilamellar lipids. Around 10–20 of the corneocytes layer ultimately form the human upper strata or SC layer, which has a variable thickness of approximately 15 microns [23]. The skin routinely permits water to transport in and out of the body. Additionally, it shows some biasness to most of the small lipophilic molecules for transport in and out of the body. Challenges occur mainly for the permeation of large size molecules, proteins, peptides, or different biologics. That is why successful commercial transdermal delivery is still confined within a short range of drug molecules.

### 2.2. Pathways of Drug Transport

**Figure 1:**
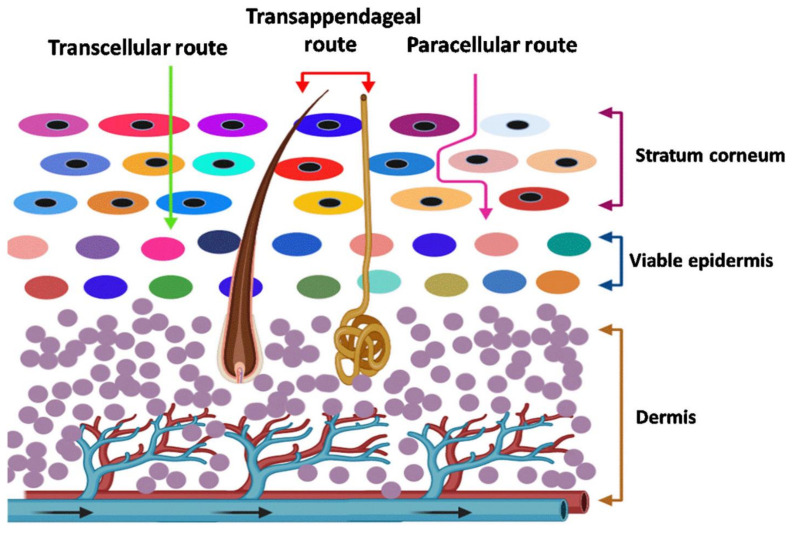
Various pathways of drug transport across the skin (Adapted with permission from [24] published by Elsevier, 2020.)

There are detailed studies and in-depth reviews on the transport mechanism of drugs by transdermal delivery. The studies revealed various drug transport pathways across the skin, which are transcellular (through the stratum corneum), intercellular (through the tight junctions of the cells), follicular or trans appendageal (sebum glands and sweat ducts) pathways [24]. In another type, by stripping or microneedling, small portions of the skin are removed to open up micro-sized pores for better drug penetration. Transdermal drug delivery can utilize the hair follicle and sebum glands as soft spots for drug penetration. Still, the appendages contribute to a minimum percentage (approximately 0.1%) of the total skin surface area [25]. Therefore, the stratum corneum is considered the targeted area of transdermal drug delivery. A summary of various pathways is presented in Figure 1.

Transcellular—Drugs are transported directly across the stratum corneum, which could be enabled by the electroporation technique.

Transappendageal—This pathway involves drug transport through hair follicles and sweat ducts. Iontophoresis and specific particulate formulations utilize this pathway.

Paracellular—This tortuous pathway transports drugs primarily within the extracellular lipids. Various permeation enhancing approaches in association with chemical, biochemical and some physical enhancers are utilized by this pathway.

### 2.3. Transdermal Permeation and Drug Characteristics

Drugs that are not well absorbed or undergoing first-pass metabolism via oral delivery are the primary target of the transdermal systems. Zero-order drug release often remains an objective of developing a transdermal system [26]. Controlled zero-order release kinetics provides a constant rate of drug release for a longer duration. In in vivo study, zero order release shows stable plasma concentration over a prolonged period. Some of the marketed transdermal products and their desirable properties are outlined in Table 1.

Log{octanol-water partition coefficient (P)}: either experimental or calculated (mean ± SD) values.

The dose is an essential concern for transdermal delivery. The majority of the transdermal products are meant for low-dose drugs (less than 20 mg) with one or two exceptions like methylphenidate patch (maximum dose 30 mg) [29]. Less potent and high-dose drugs could face problems in loading into the patch. The molecular weight of the drug is another primary concern. As shown in Table 1, marketed transdermal patches contain drugs with a molecular weight of less than 500 Da. Specific exceptions for dermatotherapy are fusidic acid (molecular weight 517 Da) and ketoconazole (531 Da) formulations. High molecular weight drugs face problems in skin permeation.

Optimizing a formulation for therapeutic effects generally implies that drug flux into the skin is maximized and obeys Fick’s first law. This requirement means that the product of drug concentration in the vehicle and drug partition coefficient between stratum corneum (SC) and vehicle be as large as possible [30]. For molecules to pass through the SC, they need to exhibit specific physicochemical properties that influence the rate of a drug’s permeation through the skin. Drugs with biphasic (water and lipid) solubility better permeate than those with high monophasic (water or lipid) solubility [31]. Highly hydrophilic drugs cannot penetrate the skin, while too lipophilic drugs have the propensity to remain in the layers of the SC. While the SC is lipophilic and favors the permeation of lipophilic drugs, the aqueous layers beneath the SC dictate that drugs should have some hydrophilic properties to pass through them. Minor structural changes of a drug, such as salt formation or esterification, can enhance aqueous or lipid solubility [3,32]. The distribution coefficient, log D value of a compound, is usually a good indication of whether a molecule would be a favorable candidate for transdermal permeation. Log D is the ratio of the sum of concentrations of a neutral and ionized compound in each of the two phases (octanol and an aqueous buffer) [33]. Log P value gives the same partition value for the neutral form of the substance.

The lipophilic nature of the SC had led to the belief that ionized drugs would be poor candidates for transdermal delivery. The transcellular route is regarded as having intermediate properties, whereas the intracellular route is mainly regarded for allowing the delivery of lipophilic molecules. Ionized drugs cross the skin through the shunt route, but the amount of molecules that pass through that route is significantly lesser than unionized molecules that take the intracellular pathway. Drugs with very low or high partition coefficients fail to reach the systemic circulation. Molecules with log P values in the range of 1–3 are considered for good permeation enhancement [24].

An increase in the number of hydrogen bonding groups of the drug may inhibit its permeation across the layers of the SC. An increase in the magnitude of hydrogen bonding causes a considerable decrease in transdermal flux [34]. On another note, an indirect relationship exists between the melting point and the solubility of a drug. Hadgraft et al. [35] developed a model that accurately estimates the solubility constraint in the SC by using the melting point. Lowering the melting point of a drug increases solubility in the SC and ultimately permeates the skin. Molecules with high melting points, due to their low solubility both in water and fat, are generally problematic in transdermal drug delivery (TDD) [36,37]. Apart from the criteria mentioned above, two other points need to consider; cutaneous metabolism of a drug that can significantly reduce its pharmacological effect and skin irritation that may reduce the patient compliance. Overall the drug properties that are favorable for transdermal delivery are summarized below;

- The dose should be less than 20 mg/day [38]
- The melting point of a drug should be less than 200 °F [38]
- The partition coefficient should be between 1 to 3 [39]
- Molecular weight < 500 Da [40]
- Non-skin irritating
- Not metabolized in skin [41]

## 3. Mechanistic Aspects of Amorphous Drug Dispersion for Transdermal Delivery

The amorphous state of drugs has higher apparent solubility and increased dissolution than its crystalline forms, improving the biopharmaceutical behavior of poorly water-soluble drugs. In several BCS class II drugs, amorphization could significantly increase the bioavailability while delivered orally. In the context of transdermal drug delivery, amorphization of drug or amorphous drug dispersion in some carriers has made its mark for better skin permeation.

Amorphous drug dispersion acts by forming a supersaturated drug reservoir on the skin. To understand the role of supersaturation in skin permeation, we should look back to Fick’s law and the theory proposed by Higuchi et al. [42].

As per Fick’s first law of diffusion, skin permeability and degree of supersaturation are correlated by Equation (1) [43].

(1) $$J=\frac{c_{v}}{c_{s,v}}.\;\frac{D.\;c_{s,m}}{L}$$

*J* is the permeation rate of a drug through skin barrier at steady-state condition, also known as steady-state flux, *c_v_* is the drug concentration in the vehicle, *c_s,v_*_,_ and *c_s,m_* are the solubility of the drug in the vehicle and the membrane, respectively. The term *c_v_*/*c_s,v_* represents the degree of saturation (DS); *D* and *L* stand for diffusivity of the drug and thickness of the barrier membrane. It is clear from the equation that if DS increases in the drug reservoir, permeation flux increases. *J* is directly proportional to the DS and independent of the concentration of the drug that requires a desired degree of saturation.

The relationship between DS and thermodynamic activity has been explained by Higuchi et al. [42]. His theory revealed that *J* (expressed as mole/m^2^s) could be correlated with a drug’s thermodynamic activity in the vehicle, expressed by *a_p_* in mole/L by Equation (2).

(2) $$J=\frac{D\;.\;a_{p}}{\gamma_{m.\;}\;.\;\;h}$$

where *γ_m_* is the activity coefficient of a drug in the skin barrier with thickness *h* (m).

Activity (*a*) and mole fraction can be expressed by the following relationship (Equation (3)),

(3) a=γ. x

where *γ* is the activity coefficient and *x* = mole fraction of the drug.

Hence, the activity can be used to define *J* instead of the partitioning of the drug in barrier membrane and vehicle. Where activity and DS can be correlated as,

(4) $$a_{p}=\mathrm{DS}=\frac{x_{p}}{x_{p}^{s}}$$

*X_p_* is the mole fraction of the drug in the vehicle, and *x^s^_p_* is the saturated solubility or mole fraction of the drug in the saturated condition in a known vehicle. It is clearly understood that as the value of *x_p_* increases, DS increases and hence the thermodynamic activity. Now Equation (2) can be rewritten as,

(5) $$J=\frac{\;D.\;a_{p}x_{m}^{s}}{h}$$

Steady-state flux is directly proportional to the thermodynamic activity, which again depends on the DS. This linear relationship is valid when the drug and vehicle or polymer do not alter the skin membrane barrier properties. In another way, it can be said that the concentration gradient is the thermodynamic force that drives the molecular movement, and diffusivity is the coefficient that expresses the diffusion kinetics [44]. The enhancement of thermodynamic activity increases with the degree of supersaturation, resulting improvement of drug diffusion. Several researchers have considered loading the drug in the supersaturated state in a patch or semisolid gel for transdermal delivery. Various methods have been undertaken to generate supersaturated drug solutions like solvent evaporation, cosolvent addition, changing pH, etc. Weng et al. (2016) have developed risperidone transdermal patch [45]. They have dissolved the drug and additives in ethanol, coated, and then evaporated the organic solvent to make a supersaturated condition. Another model lipid compound was formulated in various cosolvents like propylene glycol, ethanol, etc., to generate a two degree of supersaturation and studied the effect of DS in skin permeation [43].

Amorphization of drugs causes loss of the stable crystalline state or forms a metastable state. Amorphous or metastable drugs provide higher solubility, remain in a high energy state with higher molecular mobility and lead to supersaturation [46]. The degree of supersaturation increases with the formation of the amorphous or metastable state of the drug, and hence, drug permeation through skin increases.

## 4. Amorphous Drug Loaded Transdermal Systems

The main problem with an amorphous drug-based system is the thermodynamic instability that leads to drug recrystallization. In an amorphous state or supersaturated systems drug exists in a non-equilibrium condition and becomes thermodynamically unstable. Molecular mobility increases within the drug, which initiates nucleation followed by recrystallization [46]. Crystallization of drugs in the system reduces the degree of saturation, and subsequently, thermodynamic activity is reduced [47]. The ultimate result is the reduction of drug permeation.

Another problem is the use of organic solvents to develop an amorphous system. In most cases, ethanol, dichloromethane, or acetone are used as a solvent that needs evaporation before the final formulation of the delivery system. Higher organic solvent usage creates a negative impact on environmental sustainability,

### 4.1. Polymeric Amorphous System

Dispersing amorphous drugs in miscible hydrophilic polymers like hydroxypropyl methyl cellulose, polyvinyl pyrrolidone (PVP), grafted copolymer like Soluplus^®^ etc., is the most common approaches to inhibit recrystallization [13,48,49]. It is like solid dispersion, where amorphous drug is dispersed within a polymeric carrier when the carrier can be fully or partially amorphous. For transdermal delivery, the solid dispersion of the drug should exist as an amorphous solid dispersion containing hydrophilic polymers. These polymers act in two ways; by forming intermolecular hydrogen bonding with the drugs and filling the voids in the supersaturated solutions restricting molecular mobility. Both mechanisms help retarding the nucleation rate and recrystallization. Polymers not only help to stabilize the system but also help drug permeation from transdermal delivery. They help in a massive increase of saturation by the drug increasing thermodynamic activity. Use of other additives like acrylate polymer, Eudragit different grades like EPO, RLPO or RSPO is also used as crystallization inhibitors in the amorphous transdermal systems [50,51]. Eudragit^®^ EPO and RLPO were shown to reduce the crystallization of estradiol in a transdermal patch at 1:20 drug: Eudragit^®^ [50].

Sometimes, apart from the solubilization effect of the polymer, enhancement of solution activity causes a remarkable increase in transdermal flux. The cumulative amount of artemisin permeation through rabbit skin throughout 8 hrs was observed 5–10 times higher in the case of PVP K30 containing solid dispersion [52]. The flux enhancement ratio of solid dispersions, calculated concerning supersaturated pure drug solution, increased 5–11 folds. The study also reported an increase in flux with increasing polymeric concentration. A similar type of observation is noted on hydrophilic polymeric excipients [51]. Other aspects of the permeation enhancing effect of PVP K30 are noted. PVP tends perturbation into the hydrophobic region of the skin lipid bilayer and improves the fluidity of the region and local drug concentration [53]. In the case of Eudragit^®^, permeation enhancement has also been noted. Eudragit^®^ EPO increased estradiol permeation in ex-vivo permeation study through guineapig skin for all permeability parameters like steady-state flux, diffusivity, permeability coefficient [50]. With increasing Eudragit^®^ concentration, drug permeation was also enhanced. An increase in the rate of hydration is one cause of permeation enhancement apart from maintaining the amorphous or metastable drug state.

### 4.2. Coamorphous System

A critical issue of polymer-based amorphous drug dispersion is the adequate quantity of polymers or excipients required to prevent drug crystallization. Often it becomes high, restricting the drug loading and hence the commercial feasibility of the formulation [54]. Coamorphous system can overcome this problem. One drug is mixed with another drug or low molecular weight compound in this system, which stabilizes each other, restricts crystallization, and maintains a single-phase homogenous amorphous system. The requirement of excipient usually remains lower than the amorphous solid dispersion due to the presence of a low molecular weight compound used as coformer.

Recently few coamorphous systems have been reported where the poor soluble API is mixed with another coformer and dispersed in a viscous vehicle, often PEG 400 [15,46]. Intermolecular interaction like hydrogen bonding and ionic interaction between the two drugs or drug and coformer is the primary factor of forming a stable amorphous system. Such interactions can be evidenced by FTiR and NMR studies. In FTiR, the absence or shift in the hydrogen bond-forming group in coamorphous mixture may indicate intermolecular interaction. Acyclovir coamorphous formulation was prepared where citric acid was used as the coformer [55]. O–H peak at 3495 cm^−1^ vanished in the citric acid IR spectra while N–H stretching peak frequency was shifted to a higher side. Another O-H stretching peak was not observed in coamoprhous mixture. Such absence or shift in IR peaks is evidence of H bonding between acyclovir and citric acid. NMR analysis can be used to study the interaction between drug and conformer, where a downshift of proton (1H1) signal can be observed due to a change in electron density by intermolecular interaction [15]. Recrystallization during storage of coamorphous materials can be easily identified by powder X-ray diffraction study (PXRD), where the absence of drug crystal peaks at characteristic 2-theta angle establishes the drug amorphousness. Thermal analysis and detection of glass transition (T_g_) of the amorphous sample also indicate the recrystallization nature and storage stability. It is considered that physical instability or recrystallization would be negligible if storage temperature lies 50° below the glass transition [15].

**Figure 2:**
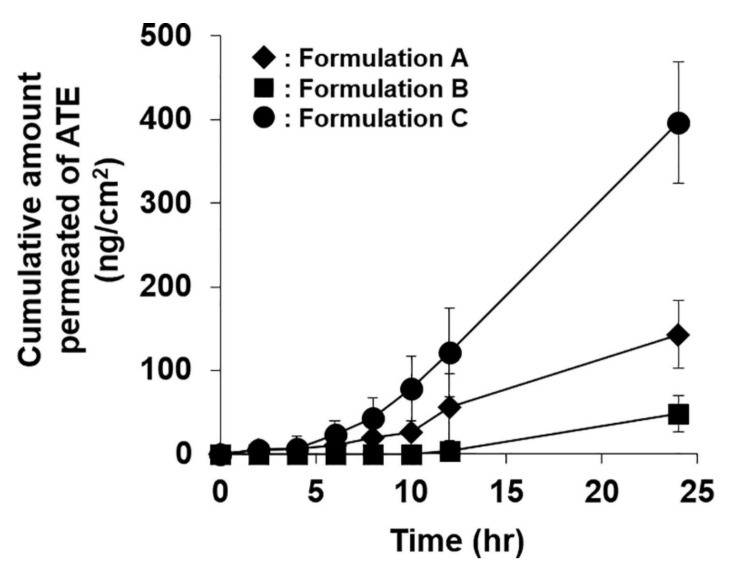
Transdermal permeation profile of atenolol (ATE) from each formulation (bars represent standard deviation, n = 4). A: pure ATE saturated suspension; B: ATE-Urea (1:8) saturated suspension; C: ATE-Urea (1:8) co-amorphous based supersaturated formulation. Figure adapted with permission from [15], Elsevier, 2019.

For skin permeation enhancement, either enhanced thermodynamic activity due to supersaturation and/or the presence of coformer (often acts as permeation enhancer) are considered significant. In some cases, coformer used in the system also acts as a permeation enhancer. Atenolol supersaturated coamrophous system was developed where urea was used as coformer [15]. Skin permeation through mice skin was observed the highest from the supersaturated coamoprhous system (Figure 2). Permeation flux of atenolol was noted 2.9 and 6 folds higher in coamorphous system than pure atenolol saturated suspension and atenolol- urea saturated suspension, respectively. Urea has played a dual role of crystallization inhibitor and penetration enhancer in this study. In another recently published study, piroxicam coamoprhous system was developed [46]. The cumulative amount of piroxicum permeated and steady-state flux through mice skin was almost doubled in coamorphous dispersion of piroxicum and citric acid (coformer) compared to a pure drug suspension and physical mixture of drug-coformer. However, in this work, the degree of supersaturation was primarily responsible for enhanced skin permeation rather than the skin penetration-enhancing property of citric acid.

#### 4.2.1. Factors to Consider for Coamorphous Transdermal System Development

Apart from the increased drug loading compared to the polymeric amorphous system or amorphous solid dispersion, the coamorphous drug-loaded transdermal system offers some more advantages. These systems have higher conformational flexibility that helps the drug and coformer to mix uniformly and prevents recrystallization. The molecular-level mixing and interaction between drug-coformer offer higher stability of the system. Small molecules used as coformer often have antiplasticizing effect, hence reducing molecular mobility [56]. The development of a transdermal coamorphous system needs critical consideration on the following factors.

##### Coformer Selection

A coamorphous system can compose two pharmacologically active molecules, which means drug-drug system or a drug with another excipient acting as coformer. In a drug-drug coamorphous system both the drugs should mix uniformly and form a glassy system. Often that does not become possible by two drugs meant for combination or synergistic therapy. The use of another excipient as a coformer is a rather more feasible approach. Various amino acids, citric acid, urea, saccharine, nicotinamide, etc. are used as coamorphous drug excipients. The selection of coformer, capable of making intermolecular interaction with the drug, is the basis for developing a stable coamorphous system. As discussed earlier, intermolecular H bonding can be identified by FTiR, NMR or raman spectra study [47,57]. DSC can study miscibility between drug-coformer. Generation of a single glass transition peak (T_g_) in DSC indicates a single-phase mixture. The selection of coformer is a complex process involving multiple analyses. There are specific theoretical approaches used to predict miscibility between various components. Solubility parameter (δ) derived from physicochemical properties and molecular weight of an element can predict the miscibility. Elements with likely values of δ are considered miscible. A difference of δ value less than 7 (MPa)^0.5^ predicts adequate [58]. The Flory-Huggins interaction parameter, derived from the melting point depression method, can also be used in molecular interaction studies between two components [59].

##### Preparation Method Selection

As discussed in the later Section 5, preparation methods can affect the structural property of coamorphous drug products, hence storage stability. Therefore, one needs to identify the suitable mode of preparation based on the thermal stability, drug-excipient miscibility, the feasibility of commercial preparation etc.

##### Selection of Vehicle

Coamorphous drugs need to dissolve in a suitable vehicle and be loaded into the delivery system, such as patch or gel. Interaction between the coamorphous drug and vehicle needs to be studied. PEG 400 is reported as a vehicle by several researchers [46,59]. The degree of supersaturation may vary in different vehicles. It needs to ensure that precipitation of drugs does not occur in the desirable vehicle. Apart from these specific factors of common factors of selecting drugs for transdermal delivery must be considered [24].

## 5. Method of Preparing Transdermal Coamorphous System

In lab scale, coamoprhous formulation can be prepared by various methods, categorized into melt-quenching, milling, solvent evaporation. In the thermal process that is melt-quenching, both drugs or drug and coformer must be heat stable. They are melted together at elevated temperatures, followed by rapid cooling. Although this method is easy, user-friendly, and there is no use of organic solvent, this method is not suitable for many drugs due to high heat stress. This method is also difficult for industrial scale. In milling, ball mill or cryo mill have been used. This technique enjoys the advantages of non-complexity, lesser chances of chemical degradation, easy to scale-up characteristics. Milling can form stable coamorphous. In cryo-mill, the milling temperature is kept very low by liquid nitrogen, allowing the technique suitable for low glass transition materials. Cryo milling, can be used as a mechanochemcial activated process and successfully applied for preparing amorphous dispersion [60]. However, chances of contamination, time and labor consumption, percentage loss, limited capacity of amorphization compared to the other two methods are the disadvantages of the ball milling method.

Solvent-based methods are the third category that could be suitable for heat-sensitive drugs. In this method, drug-coformer mixture is dissolved in a vehicle, primarily organic, and then dried by spray drying or vacuum drying. This method is effective for converting the crystalline drug to amorphous. Spray drying is also a widely used and adopted technique in the industry. So the scalability of the solvent evaporation method is adequate. The product comes as free-flowing powder, which is attractive for further downstream processing. In a recent study, Ruponen et al. [61] have formulated large scale furosemide- arginine coamoprhous where no sign of crystallinity was noted in the formulation. However, selecting a common solvent for both of the components often becomes a challenge to the formulation scientist. There is a negative environmental impact of high organic solvent use in large-scale manufacturing. Lastly, residual solvents present in the product may initiate crystal growth during storage.

**Figure 3:**
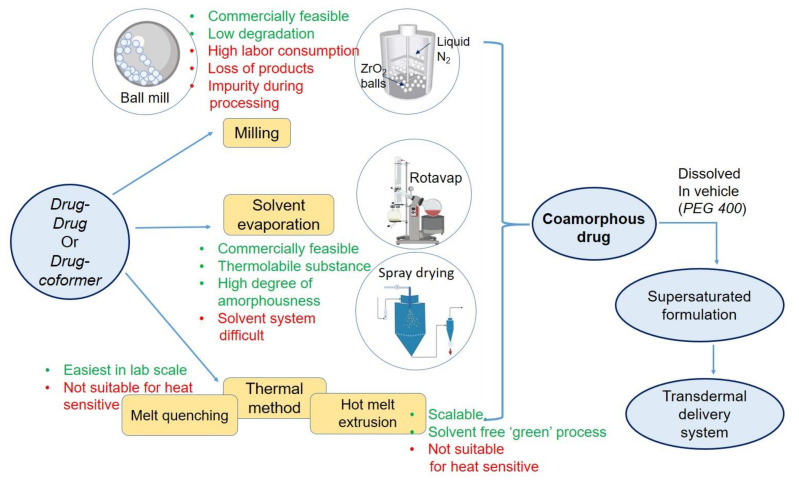
Various method of formulating coamorphous drug loaded transdermal system.

At present, melt extrusion method, named hot-melt extrusion (HME), a thermal method, is gaining popularity for the processing of coamorphous products. In the latter section, the implementation of HME technique for transdermal amorphous formulation has been discussed. Preparation methods and their process variables affect the structural property and final product characteristics significantly. In a study by Lim et al. [62], thermal method, dry milling, and solvent-based methods were compared to prepare cimetidine-indomethacin and cimetidine-naproxen coamorphous. Coamorphous drugs, prepared by all methods, retained their amorphousness over six months of storage at 40 °C. However, a mechanistic study found higher molecular mobility in both coamorphous prepared by dry ball milling or cogrinding of both drugs. Therefore, the selection of a suitable method is very critical for preparing coamorphous system. An overall summary of coamorphous preparation methods is presented in Figure 3.

While solvent free methods of preparing amorphous drug formulation are in demand, HME has emerged as a possible alternative. HME is considered a ‘green’ approach in industrial applications. Apart from no use of organic solvents, continuous processing one-step manufacturing are some of its advantages. In pharmaceutical industries, HME is widely used to successfully apply various formulation approaches like cocrystals, ASD, melt granulations, co-extruded formulation, micronized system, 3D printing, and coamorphous systems, polymeric film, etc. [63]. Broadly, this equipment consists of a barrel and an extruder. The barrel consists of various zones, providing the formulator the flexibility of adding materials in any zone and playing with a different temperature range. Such flexibility helps the formulator optimizing the residence time of certain materials in a particular zone controlling the heat exposure of drug and other excipients. Different features and applications of HME have been reviewed recently by various other researchers [63,64]. HME’s application has largely propagated to formulate ASD and to enhance oral bioavailability of poorly water-soluble drugs, mainly BCS class II [65,66]. This technique’s clinical and commercial success is indicated by the presence of several melt-extruded ASD products in the market [66].

HME method has been applied to develop several coamorphous formulations with or without polymeric stabilizer. Lenz et al. (2017), reported coamorphous formation of indomethacin with arginine coformer and a polymer [67]. For transdermal application, HME has been applied to formulate carvedilol containing extrudate based gel with soluplus^®^ and cyclodextrin [68], eudragit RS PO based extruded film [51], etc. A ketoprofen transdermal gel is also reported where amorphous dispersion of ketoprofen was obtained in poloxamer carrier by HME technique [69]. In all cases, enhancement of skin permeation and retaining drug amorphousness have been reported. The outcome of these researches is presented in Table 2. Drugs in molecular dispersion increases effective concentration and hence thermodynamic activity, resulting enhanced skin permeation. A sustained zero order drug diffusion through skin is also observed. Despite several advantages, high processing temperature, not much availability of suitable polymers is some of the challenges that HME still needs to overcome.

## 6. Clinical/Preclinical Studies and Patents with Amorphous Drug Based Transdermal System

Clinical trial search on the transdermal system from Clinical Trials registry revealed 478 study titles, which used ‘transdermal’ keyword [70]. However, the total number of molecules studied under the trials is limited to not more than ten. The maximum number of trials examined nicotine, fentanyl, estradiol, buprenorphine, testosterone, rotigotine, rivastigmine, etc. The lesser number of therapeutic molecules under trials are in line with the lesser number molecules commercialized as transdermal product. Watkinson has reviewed the classes of drugs undergone clinical trials as well as marketed [71]. The author explained that smaller molecules with considerable log P value and high potency were only the target for successful transdermal delivery. Amorphous drug-loaded transdermal systems have not been studied clinically. Major problem associated with such system is the stability of the amorphous drugs. Loading of an amorphous dispersion into transdermal patch requires some further steps, which include solvent evaporation or long exposure to environmental condition. These steps might initiate the recrystallization of drugs. Once the amorphous nature is lost, the supersaturation characteristics would be compromised. Moreover, polymeric amorphous dispersion contains higher amount of polymeric carrier, which restricts the drug loading into transdermal patch. This is the major reason behind the lacking of clinical studies of amorphous dispersion loaded transdermal patch.

While solvent evaporation-based supersaturated transdermal systems are studied for in vivo parameters, preclinical in vivo or pharmacokinetic evaluation with polymeric amorphous or coamorphous transdermal systems have not been reported. Most of the studies are restricted to ex-vivo skin permeation or skin uptake studies. Ex-vivo skin permeation study provides an idea on the potential of transdermal formulation for uptake of the drug, its deposition in the skin, and systemic absorption. But, ex-vivo skin permeation study should be correlated with in vivo pharmacokinetics and their desired therapeutic responses in animal models. Several inventions on transdermal delivery system have been published or protected by patents. However, transdermal drug delivery based on the amorphization of drugs has not been patented exclusively. Only a few patents have been published depicting such inventions. A summary of the relevant patents is presented in Table 3.

## 7. Future Direction

Amorphous drug-loaded supersaturable system can be a promising approach in transdermal drug delivery shortly. But imparting stability (prevention of recrystallization) would be a significant hurdle. The future development of transdermal drug delivery with the drug amorphization concept would be directed towards a stable transdermal patch. Stable polymeric amorphous systems can be prepared, but the drug load becomes low. Therefore, the direction of research would be developing a new carrier system for improving drug load. A porous carrier such as mesoporous silica with high drug loading capacity with the ability to prevent nucleation of the loaded drug is an upcoming interest. Industrially feasible HME method is already adopted for processing amorphous systems. But the application of melt-extruded products for transdermal delivery is to be explored. The future development in this field could be the implementation of HME based amorphous drug-loaded transdermal gel. But finding suitable polymers with adequate glass transition temperature would be a prerequisite. Coamorphous systems should optimize the drug- coformer ratio targeting high drug loading and better stability.

Loading the coamorphous system into the suitable vehicle and converting it to the final applicable dosage form, either patch or gel should be of significant interest. Coformers like urea, citric acid can absorb moisture easily if exposed to the open environment. Therefore processing of such coamorphous system requires special attention. It is essential to study the stability of the amorphous drugs after dispersing them into suitable carriers, followed by loading them into the patch. Future studies with coamorphous transdermal systems should focus on in vivo studies. The systemic availability and the concentration of drugs in the blood vs. time profile must be developed. Such a profile can determine how long the drug concentration remains above the minimum effective concentration for a therapeutic response. Then, the controlled and sustained drug release can be justified.

## 8. Conclusions

Overall mechanisms of drug permeation through skin have been studied by this review article. A glimpse of various approaches of promoting drug transport overcoming the skin barrier has also been given. Supersaturated drug systems have a higher thermodynamic activity which helps their diffusion through the skin barrier. Polymeric amorphous systems and coamorphous systems both possess a higher degree of supersaturation, hence providing better drug permeation. Prevention of drug recrystallization is the main target of both systems. However, a detailed stability study as per the regulatory guidelines and in vivo pharmacokinetics and pharmacodynamic studies can provide more in-depth knowledge required to further the amorphous drug-loaded supersaturated transdermal delivery system.

## Figures and Tables

**Table 1:** Marketed transdermal formulations and associated properties ([27,28]).

Transdermal Drug	Year	Molecular Weight (g/mol)	Oral Bioavailability (%)	Log P_ow_	Dose/Day (mg)
Scopolamine	1979	303.35	27	0.98	0.3
Clonidine	1984	230.09	65	2.42 ± 0.52	0.1–0.3
Fentanyl	1990	336.47	92	4.05	0.288–2.400
Nicotine	1991	162.23	30	1.17	7–21
Testosterone	1993	288.42	<1	3.32	0.3–5
Lidocaine	1995	234.34	<1	2.84	
Norelgestromin & Ethinyl estradiol	2001	327 & 296.40	40–43	3.90 ± 0.47 3.67	0.2 & 0.034
Oxybutynin	2003	357.49	6	4.02 ± 0.52	3.9
Methylphenidate	2006	233.31	5–20	2.15 ± 0.42	10–30/9 h
Selegiline	2006	187.28	4–10	2.7	6–12
Rotigotine	2007	315.47	<1	4.58 ± 0.72	1–3
Rivastigmine	2007	250.34	40	2.34 ± 0.16	4.6–9.5
Granisetron	2008	312.41	60	2.55 ± 0.28	3.1
Buprenorphine	2010	467.64	10–15	4.98	0.12–0.68

**Table 2:** Transdermal amorphous drug-loaded delivery system formulated by HME.

Name of the Drug and Type of the Formulation	HME (Polymer and Processing Condition)	Outcomes	Discussion
Indoemthacin-arginine coamorphous [67]	Components: Copovidone (20%) Indomethacin (IND) 53.8% Arginine (ARG) 26.2%, Extruder temperature: 70 °C, Speed at 50 RPM for 20 min. The product was a viscous yellowish gel.	Stable coamorphous product No sign of crystallinity upto 6 months of storage in at 23 °C or 40 °C over silica gel. An immediate and high supersaturation (cmax about 101 mg/L) in in vitro dissolution	Ionic interaction between copovidone and IND-ARG occurred Amorphousnesss of ARG is maintained by copovidone. Ex-vivo or In-vivo study was not reported.
Ketoprofen gel [69]	Components: Ketoprofen (KTP) was extruded by a Twin screw extruder containing poloxamer 407 (30–40%). Temperature range for various zones of the extruder were 97 to 70 °C, screw speed at 50 RPM.	For 40% poloxamer gels, the cumulative amount of drug permeated/unit area of the porcine epidermis from the extruded gel was 2.86 ± 0.31 μg/cm^2^ compared to 1.54 ± 0.27 μg/cm^2^ from the control gel.	KTP was converted to fully amorphous form in the gel. Supersaturated state of the drug promoted the skin permeation with enhanced steady state flux.
Carvedilol supramolecular gel [68]	Components: Carvedilol, either α- cyclodextrin or β-cyclodextrin, Soluplus^®^ and PEG 400 Twin screw extruder at a temperature gradient of 150/160/170 °C, speed at 150 rpm.	No drug melting event was recorded at 117.4 °C. Supramolecular gel showed the lowest 47.75% and the highest 80.49% of in vitro drug release from α cylcodextrin and β cyclodextrin, respectively.	HME induced amoprhization favoured carvedilol to form inclusion complex with cyclodextrin. Molecular level dispersion and amorphization induced supersaturation occurred in the gel.

**Table 3:** Patents on transdermal amorphous drug loaded system.

Patent No	Title, Filed/Approved Year	Invention Detail
US20130316996A1 [72]	Transdermal delivery rate control using amorphous pharmaceutical compositions, 2014	A transdermal delivery system where amorphous drug deposits are formed in situ, in the stratum corneum due to solvent evaporation. The composition is claimed 0.1% to about 10% of the drug or active agent, from about 0.1% to about 10% of a non-volatile dermal penetration enhancer, and from about 85% to about 99.8% of the volatile solvent by weight. A zero order drug release has been achieved for maximum 10 h.
US9925150B2 [73]	Polyvinylpyrrolidone for the stabilization of a solid dispersion of the non-crystalline form of rotigotine, 2018	A polymeric amorphous solid dispersion system for transdermal drug delivery of rotigotine with Polyvinylpyrrolidone (PVP) as polymeric stabilizer. The ratio of rotigotine: PVP has been varied from 9:3.5 to about 9:6 (*w*/*w*). Rotigotine free base and PVP forms ‘microreservoir’ in a suitable dispersion medium.
EP252233 3 [74]	Amorphous drug transdermal systems, manufacturing methods, and stabilization, Published in 2014	A transdermal supersaturated system of oxybutynin. The system comprises of a backing layer, and an adhesive layer in which amorphous active agent is dispersed and a release liner.
